# Hybrid mini-thoracotomy for Brugada syndrome: epicardial substrate characterization and ablation—results from UNCOVER(BrS) study

**DOI:** 10.3389/fcvm.2025.1658355

**Published:** 2025-10-28

**Authors:** Saverio Iacopino, Paolo Francesco Sorrenti, Andrea Petretta, Jacopo Colella, Alessandro Di Vilio, Giovanni Statuto, Gennaro Fabiano, Giuseppe Campagna, Gianluca Peluso, Emmanuel Fabiano, Giuseppe Indellicati, Simona Brogneri, Elena Tremoli, Lorenzo Mantovani, Giuseppe Speziale, Carlo Savini, Alberto Tripodi

**Affiliations:** ^1^ Arrhythmology Department, Maria Cecilia Hospital, Cotignola, Italy; ^2^ Università Magna Graecia, Catanzaro, Italy; ^3^ San Carlo di Nancy, Roma, Italy

**Keywords:** Brugada syndrome, catheter ablation, mini-thoracotomy, epicardial substrate, omnipolar mapping

## Abstract

**Background:**

Data on the safety and efficacy of thoracotomy epicardial ablation for Brugada syndrome (BrS) are limited. The ongoing UNCOVER(BrS) trial aims to evaluate epicardial substrate homogenization in patients with symptomatic BrS.

**Objective:**

To report the study design and preliminary outcomes of a novel hybrid mini-thoracotomy approach for Brugada substrate mapping and ablation in an initial cohort of patients with BrS.

**Methods:**

This independent, prospective pilot study was conducted at a single center. Patients with BrS who had an implantable cardioverter-defibrillator (ICD) and a history of symptomatic events (syncope and/or documented ventricular arrhythmia) were selected for epicardial ablation. Following surgical access via mini-thoracotomy, substrate mapping was performed using the Advisor™ HD Grid catheter to identify the pathological substrate after an ajmaline infusion. Point-by-point ablation with a contact force catheter was performed to achieve substrate homogenization.

**Results:**

Between January 2022 and July 2024, six patients were enrolled. No major acute peri-, or post-procedural complications were observed. The procedure acutely eliminated all pathological substrates, with complete suppression of the Brugada ECG pattern. Procedural efficiency improved over time, with a progressive reduction in procedural duration. At the 12-month follow-up, none of the patients had any symptomatic arrhythmic events. Omnipolar mapping demonstrated superior delineation of the arrhythmic substrate compared to bipolar mapping.

**Conclusion:**

Epicardial substrate ablation via a hybrid mini-thoracotomy approach was found to be technically feasible and preliminarily safe in this initial assessment. No acute or long-term major adverse events were observed. By the 12-month follow-up, no symptomatic arrhythmic episodes had occurred. Larger studies with extended follow-up durations are needed to validate these initial findings.

**Trial registration:**

ClinicalTrials.gov identifier: NCT05643209. Funded by Abbott Medical.

## Introduction

Patients with symptomatic Brugada syndrome (BrS) with a history of aborted sudden cardiac death or arrhythmic syncope are at a higher risk for recurrent ventricular arrhythmias (VAs) in the absence of structural heart disease (1–4). Risk stratification remains challenging, as the majority of patients are asymptomatic at the time of diagnosis (5, 6). Programmed ventricular stimulation (PVS) plays a key role in the complex decision-making process for managing patients who are considered to be at lower risk (7).

The Italian Brugada Syndrome (IBRYD) registry showed that patients with a drug-induced type I Brugada ECG pattern have an increased risk of arrhythmia (1.2%/year), supporting the use of PVS during the risk assessment (8). Electrocardiographic markers are emerging as promising tools for risk stratification. Among them, the novel dST-Tiso interval >300 marker has been proposed as a predictor of VA inducibility in patients with a drug-induced type I ECG patterns, with 75% sensitivity and 86% specificity, and it is speculated that a 2% increased risk of inducibility is observed for each 1 ms increase in dST-Tiso duration (9, 10).

Implantable cardioverter-defibrillator (ICD) therapy is indicated for secondary prevention in symptomatic patients due to their elevated risk of recurrent arrhythmia (1). The current guidelines recommend catheter ablation of the right ventricular (RV) outflow tract’s (RVOT) epicardial substrate in patients with BrS using appropriate recurrent ICD shocks refractory to drug therapy (Class IIa) (1). The technique introduced by Nademanee et al. (11) focuses on accurate identification and complete elimination of the abnormal electrical potential induced by sodium channel blockers localized in the epicardial substrate of the RVOT (12–14). Ablation of these regions has been shown to significantly suppress recurrent ventricular fibrillation and normalize the ECG in over 75% of patients (15). Various substrate mapping and ablation strategies have been proposed, the majority of which involve epicardial access via a subxiphoid puncture, with mapping performed using either a standard linear diagnostic or ablation catheter (12–17).

A hybrid thoracoscopic epicardial RVOT ablation approach was introduced by Salghetti et al., demonstrating the safety and the feasibility of the technique, which allows for direct visualization of ablation during radiofrequency delivery (18). Recently, our group reported successful epicardial substrate ablation in BrS using a novel hybrid mini-thoracotomy approach (19, 20).

The limited resolution and larger electrode size of conventional catheters affect the detection of abnormal, prolonged, fragmented bipolar electrograms (BiEGMs), which are commonly observed on the RVOT’s epicardial surface. High-density mapping with omnipolar technology electrograms (OTEGMs) has recently enabled more accurate identification of the pathological BrS substrate, with direct wavefront visualization enhancing the delineation of deceleration zones and block lines (19). Similarly, in thoracoscopic BrS ablation, the integration of a 3D mapping system and high-resolution grid catheters improves substrate identification (21). Long-term follow-up data after ablation remain limited, particularly for hybrid approaches.

This study aims to investigate the technical feasibility and the initial efficacy of a hybrid mini-thoracotomy approach for BrS substrate ablation. Substrate identification will be performed with equi-spaced electrodes and OTEGMs, targeting abnormal, prolonged, fragmented low-frequency ventricular electrograms. We also aim to identify the most appropriate mapping setting to describe the conduction properties of the arrhythmogenic substrate in BrS.

## Methods

### Trial design and patients

This was an independent, single-center, prospective study that was designed to evaluate the feasibility, efficacy, and safety of a hybrid thoracotomy approach with epicardial substrate mapping and ablation in patients with BrS. Herein, we report the results of the first six patients enrolled in the UNCOVER(BrS) study (NCT05643209), which was conducted at Maria Cecilia Hospital (GVM Care & Research; Cotignola, Italy). The study was approved by the local ethics committee, and written informed consent was obtained from all patients.

Patients diagnosed with a spontaneous type-1 Brugada ECG pattern, induced after an ajmaline infusion, and who had undergone an ICD implantation were screened for symptomatic events. After ICD implantation, patients with syncope events and/or documented ventricular arrhythmia were considered symptomatic. Eligible patients were selected for ablation with a 3D high-density mapping system in accordance with current guidelines and medical decision-making, and they were enrolled in the study after providing informed consent. The exclusion criteria included the inability to provide written informed consent, the inability to comply with follow-up visits, a life expectancy of less than 12 months based on clinical assessment, prior cardiac ablation within 90 days before enrollment, and pregnancy.

### Study endpoints

The primary endpoint was the survival-free rate of symptomatic events related to episodes of ventricular arrhythmia at the 6- and 12-month follow-ups after the procedure. Symptomatic events were defined as documented episodes of ventricular arrhythmia and/or episodes of arrhythmic syncope.

The secondary endpoint was to identify the most appropriate mapping configuration to describe the conduction properties of the arrhythmogenic substrate in BrS. Data from late potentials and voltage maps, acquired using different settings (BiEGM vs. OTEGM), conduction vectors, and wave speed maps, were analyzed to provide a detailed electrical characterization of abnormal myocardial tissue.

### Hybrid mini-thoracotomy procedure

**Figure 1:**
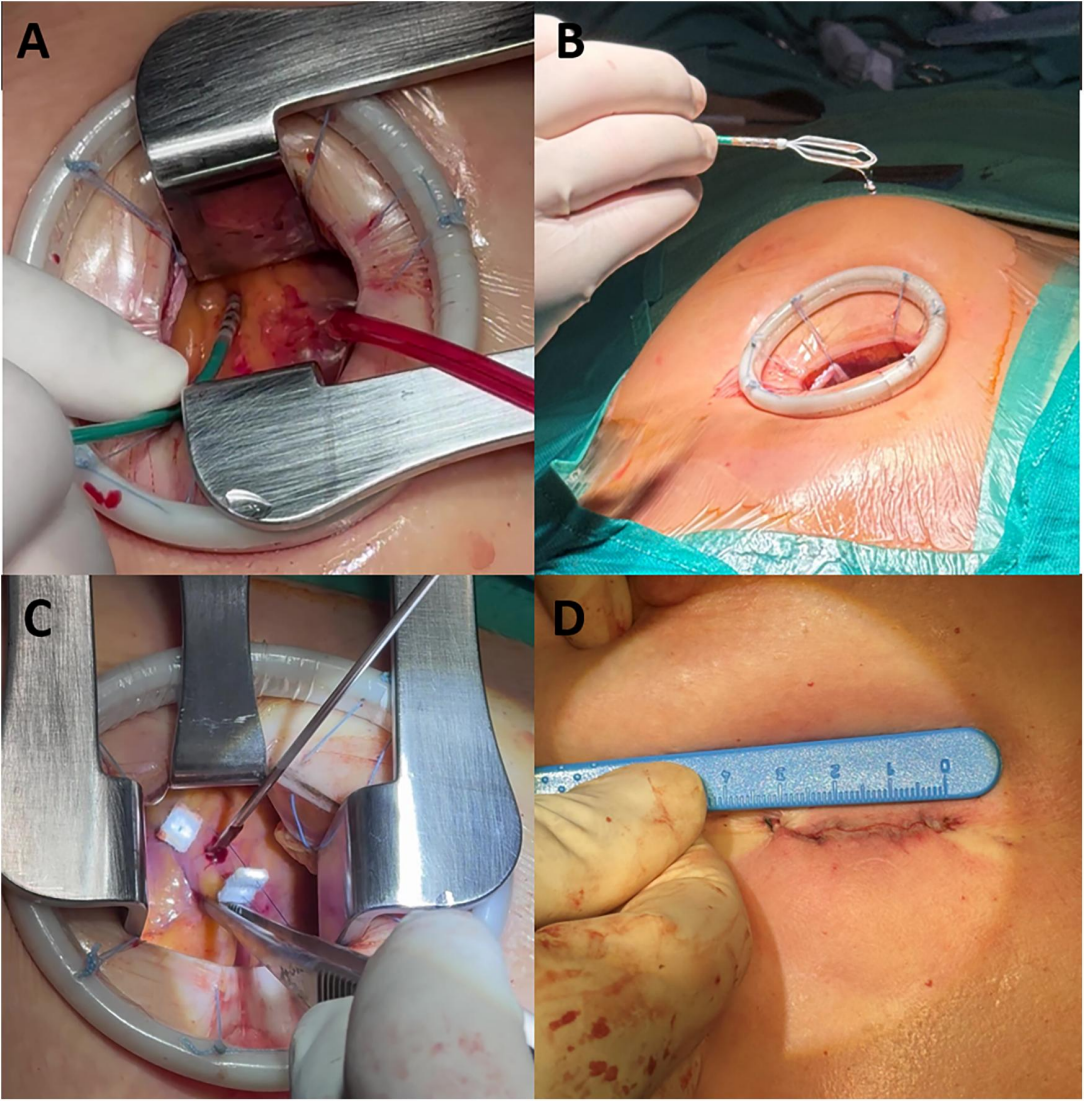
The hybrid mini-thoracotomy approach. **(A)** Ablation catheter manipulation through the mini anterior thoracotomy access. **(B)** Advisor HD Grid mapping catheter manipulation. **(C)** Epimyocardial biopsy sample collection. **(D)** Residual surgical wound at the end of the procedure.

The hybrid mini-thoracotomy approach for epicardial ablation in patients with BrS has been previously described (19, 20). Briefly, pericardial access was obtained by a cardiac surgery team via left anterior mini-thoracotomy, with the patient in a supine position under general anesthesia. A 3–4-cm skin incision was made either over the third left intercostal space or periareolarly. The subcutaneous tissue and muscle layers were then dissected using electrocautery until the upper edge of the rib was reached. After dividing the external and internal intercostal muscles, ventilation of the left lung was temporarily suspended, and a tissue retractor was used to displace the lung, allowing direct visualization and access to the pericardial space above the RVOT. Epicardial mapping and ablation were performed by the electrophysiology team. The catheters were introduced through the thoracotomy access. The open surgical field enables direct visualization of the anterior RV wall, allowing the operator to manipulate the catheters manually using their fingers or forceps (Figure 1).

Initial mapping was performed using a contact force catheter (TactiCath™ or TactiFlex™ Sensor Enabled™, Abbott, Chicago, IL, USA) in Voxel mode in the mapping system (EnSite™ X, Abbott, Chicago, IL, USA) to delineate the epicardial surface boundaries. Next, high-density mapping was performed using a multipolar grid catheter (Advisor™ HD Grid Sensor Enabled™, Abbott, Chicago, IL, USA) during sinus rhythm to acquire voltage and potential duration bipolar maps of the anterior epicardial substrate of the RVOT. Automatic software annotation (last deflection) was used and manually verified.

Following an ajmaline infusion (1 mg/kg over 5 min), a second high-density map was acquired to identify the BrS substrate. Abnormal signals were evaluated based on voltage amplitude, fractionation, signal duration, and morphology. Substrate analysis aimed to identify conduction block zones and areas of electrical dispersion. The identified pathological area was tagged and delimited for ablation.

Substrate homogenization was performed using a contact-force ablation catheter with a point-by-point technique, applying radiofrequency energy at a power limit of 40 W and a maximum tip temperature of 43°C. The target inter-lesion distance was 6 mm, with each lesion delivered for 20 s under stable contact. RF applications were delivered over the entire pathological area previously identified and delineated in the mapping system.

Post ablation, ajmaline was re-administered to confirm complete substrate elimination. Any residual abnormal potential was targeted with additional RF applications until complete substrate abolition was achieved.

### Follow-up

The patients remained hospitalized for at least 3 days post-procedure for monitoring purposes. Before discharge, clinical (vital signs and therapy adjustments) and instrumental evaluations (12-lead ECG and echocardiography) were performed. Follow-up visits were scheduled at 3, 6, and 12 months, assessing symptoms, adverse events, vital parameters, 12-lead ECGs, echocardiograms, 24 h Holter monitoring, and ICD interrogation. The ajmaline test was repeated at 6 and 12 months to evaluate for any recurrence of the Brugada ECG pattern. No additional invasive electrophysiology studies were performed during follow-up.

### Statistical analysis

Continuous variables are presented as a median and interquartile range due to the small sample size. Continuous variables were compared using the Mann–Whitney *U* test and the Wilcoxon signed-rank test. Categorical variables are expressed as a frequency or percentage. Categorical variables were compared using the chi-squared test. *P*-values  < 0.05 were considered statistically significant. The statistical analysis was performed using SPSS V.22.0 (IBM).

## Results

### Patient population

**Figure 2:**
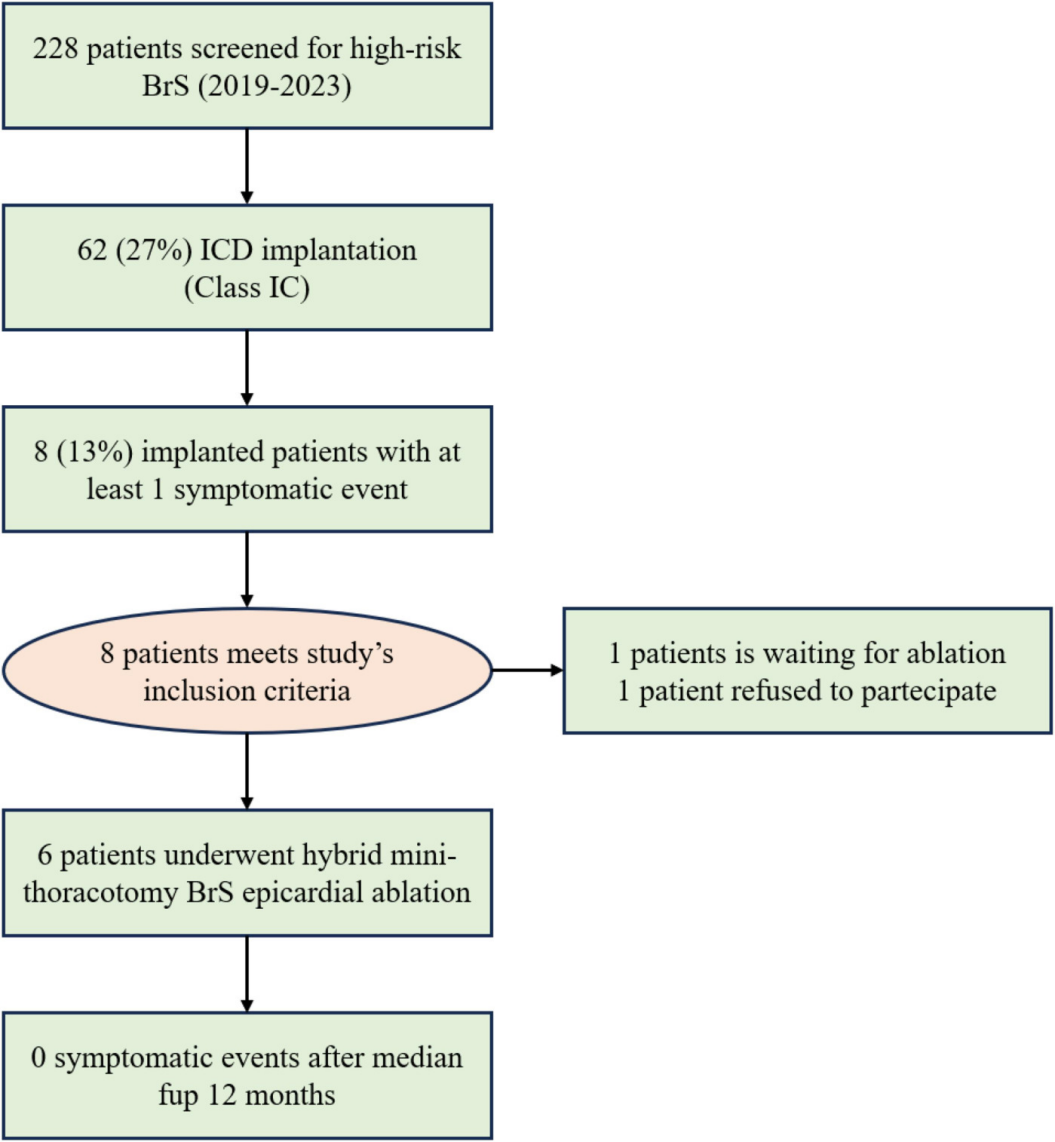
A flow chart of the study illustrating the evolution of the BrS population. In total, 13% of the implanted patients experienced a symptomatic event and were included in the study.

A total of 228 patients were screened for BrS. Of these, 62 (27%) underwent ICD implantation (Class Ic^1^) and were monitored for symptomatic episodes. Eight patients experienced at least one symptomatic event, including syncope and/or ventricular arrhythmia. Six patients consented to and underwent catheter ablation (Figure 2; Supplementary Table S1).

Two patients received subcutaneous ICDs (S-ICDs) and four patients received transvenous ICDs. The median age at ICD implantation was 53 years (range: 34–70). ICD implantation followed a comprehensive evaluation for a characteristic type 1 BrS ECG pattern, which was either spontaneous or unmasked during an ajmaline infusion, and risk stratification for arrhythmia.

Programmed ventricular stimulation was performed as part of the risk stratification process. Inducible VA with two extra stimuli was observed in three patients (patients #3, #4, and #5). One patient (patient #5) carried a pathogenic *SCN5A* gene mutation. Two patients (patients #1 and #2) had a family history of BrS and had experienced documented episodes of polymorphic ventricular tachycardia during 7-day Holter ECG monitoring, although arrhythmia was not inducible during PVS.

All patients exhibited a type 1 ECG pattern during the ajmaline infusion, with a prolonged dST-Tiso interval >300 ms [median: 320 ms (IQR: 310–340 ms)].

### Efficiency of the hybrid thoracotomy procedure

**Figure 3:**
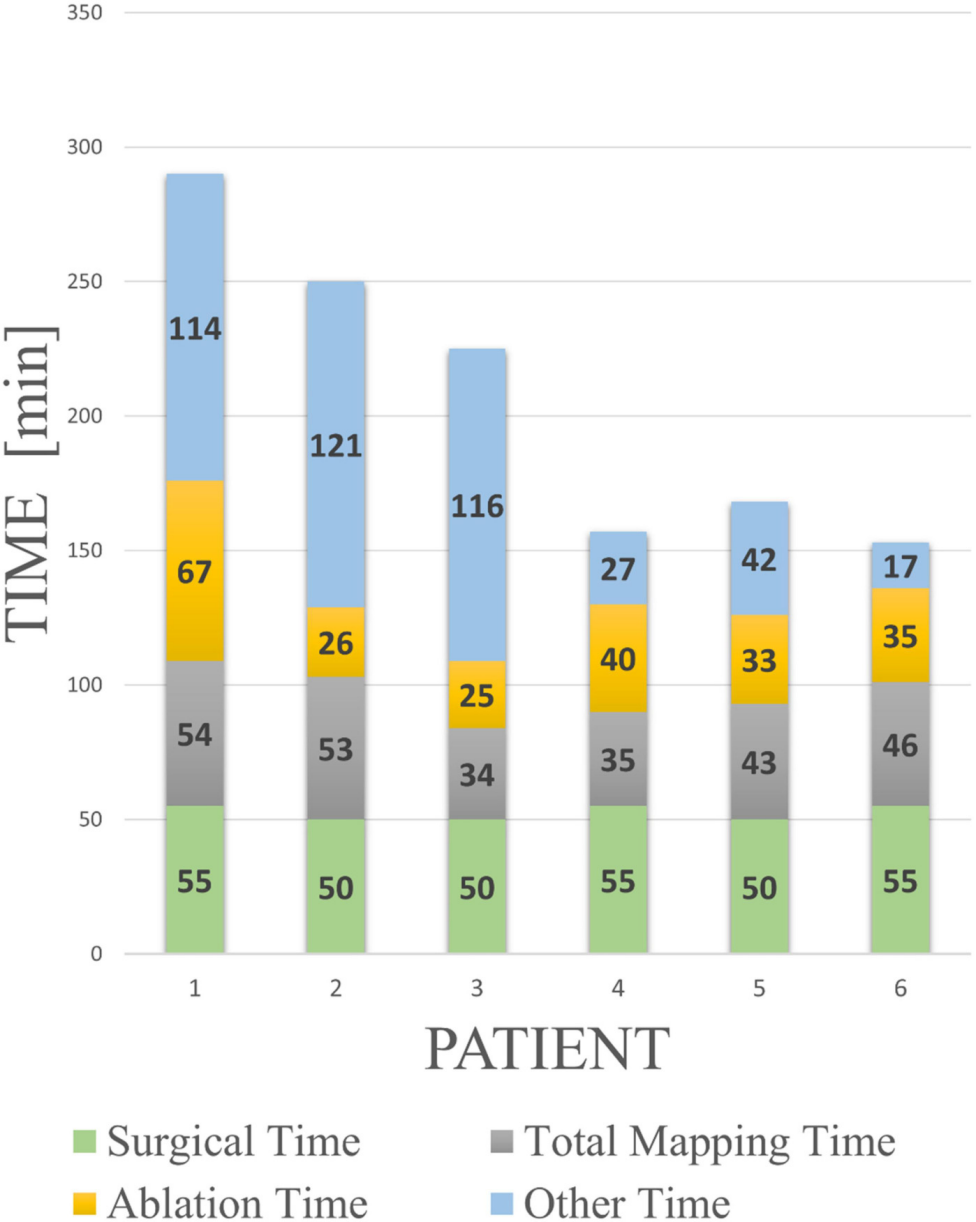
Procedural learning curve with a breakdown of the procedural times. Surgical time refers to the time taken by the thoracic surgeon to open and close the thoracic window. Total mapping time includes Voxel collection, baseline mapping, ajmaline mapping, and all post-RF application mapping performed during the validation phase. Ablation time spans from the first to the last RF application. “Other time” includes device preparation, biopsy sample collection, ajmaline infusion, and troubleshooting operations. The learning curve is especially evident in the progressive optimization of the latter procedural components.

All patients underwent epicardial mapping and ablation via a mini-thoracotomy approach. The median skin-to-skin procedure duration, including surgical time, was 200 min (IQR:157–260) (Figure 3). Surgical time was consistent across cases [median: 50 min (IQR:50–55)], whereas the electrophysiological operating time progressively decreased [median: 150 min (IQR:115–225)].

### Epicardial substrate characterization

All patients underwent direct epicardial mapping under baseline conditions, after an ajmaline infusion, and following RF application.

**Figure 4:**
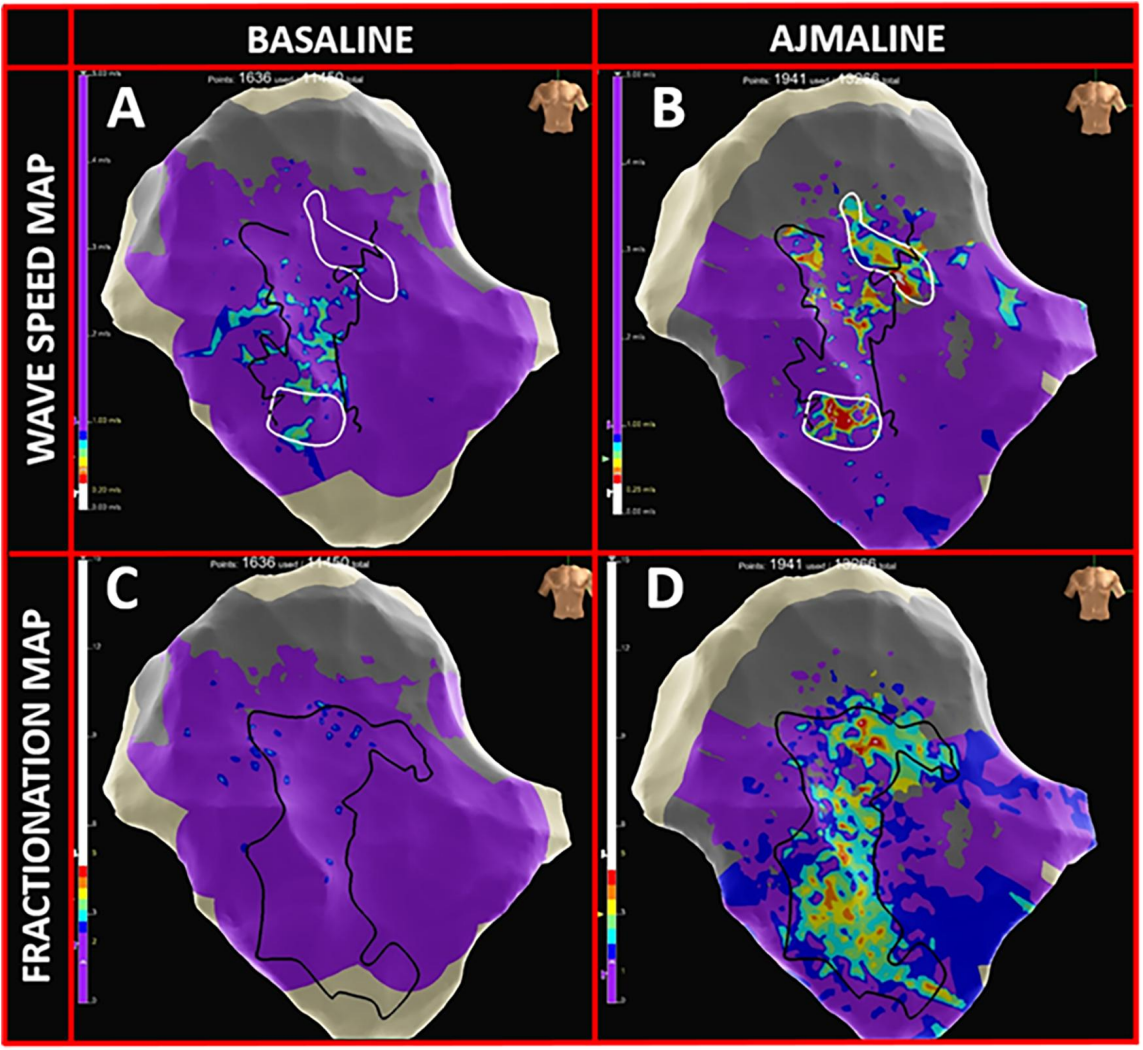
High-density omnipolar mapping features. **(A)** Wave speed map in baseline conditions with >90% epicardial velocity conductions >1 mm/ms (purple). **(B)** Appearance of multiple delayed conduction zones (rainbow colors; ranging from 0.25–0.5 mm/ms) in proximity to BrS substrate borders (dark lines). **(C)** Fragmentation map at baseline, prior to the ajmaline infusion, with >95% of epicardial OTEGMs with <2 fragmentations (purple). **(D)** Appearance of highly fragmented signals (rainbow colors; 2 to 5 deflections) inside the BrS pathological substrate following the ajmaline infusion.

In the baseline conditions, patchy low-voltage areas (<1 mV) were identified in 50% of patients with a median extension area of 4.0 cm^2^ (IQR: 2.2–7.6). Late potentials (outside the QRS complex) were observed in an area of 0.5 cm^2^ (IQR: 0–3). The isochronal late activation mapping (ILAM) showed no deceleration zones (defined as >3 color bands per linear cm), and no conduction blocks were present. The fragmentation maps revealed no signals with >2 components (Figure 4C), and the wave speed maps demonstrated normal conduction velocities (>1 mm/ms) across the epicardial surface (Figure 4A).

Following the ajmaline infusion, all patients exhibited one or more areas with late, fragmented, long-lasting multicomponent electrograms with a median extension of 10.3 cm^2^ (IQR: 3.7–12.5). Signal fragmentation increased by up to five deflection components in the fragmentation maps (Figure 4D). Moreover, while under the effect of the drug, conduction block lines emerged, replacing the previously linear propagation pattern. Deceleration zones appeared on the ILAM maps, and the wave speed maps revealed localized zones of slowed conduction (<1 mm/ms; rainbow-colored zones in Figure 4B).

**Figure 5:**
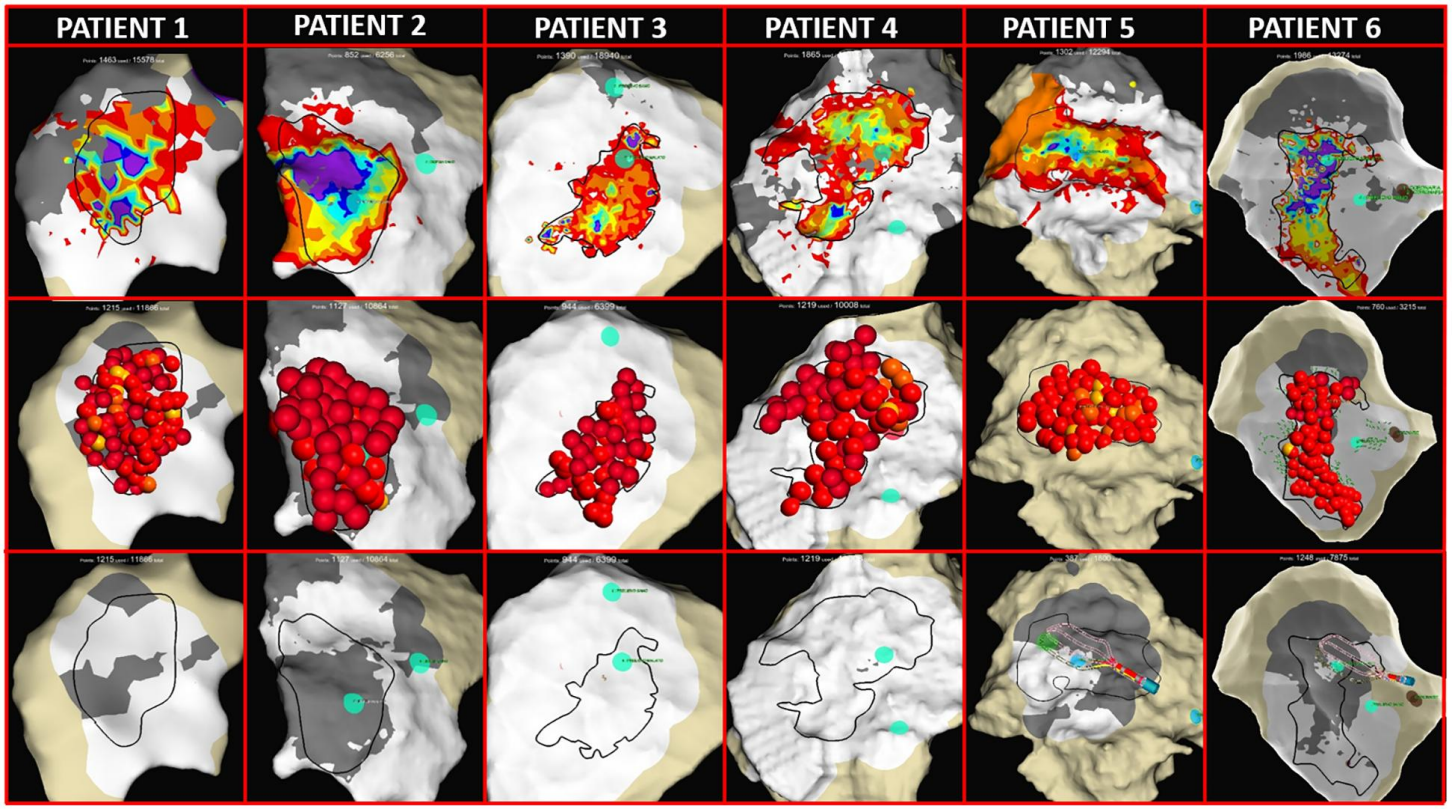
Thoracotomy and epicardial mapping and ablation. (First row) LAT maps from all patients showing the pathological prolongations of OTEGMs after the ajmaline infusion (rainbow colors). The dark lines identify the target area for ablation. (Second row) EnSite maps with red 3D dots representing RF application on the epicardial surface. (Third row) LAT maps obtained after RF ablation and the ajmaline infusion showing complete BrS substrate homogenization with no fragmented prolonged potentials (white areas) and the appearance of scar areas (gray areas indicate <0.1 mV).

Pathological substrate identification was based on an OTEGM LAT map analysis, targeting regions with long-lasting fragmented signals, deceleration zones, and block lines post-ajmaline. The median area targeted for RF ablation was 6.6 cm^2^ (IQR: 4.2–8.9). Figure 5 illustrates the ablation target zones (dark lines) overlaid on LAT maps showing prolonged electrograms.

### Pathological substrate ablation

Point-by-point RF applications were delivered at 40 W for a median duration of 14 min (IQR:10–17) to achieve full pathological substrate elimination (voltage reduction <1 mV) over an epicardial area of 11 cm^2^ (IQR: 8–15) (Figure 5). Due to direct catheter manipulation close to the ablation tip, a median contact force of 19 g (IQR: 13–30) was achieved (Supplementary Table S2).

Following RF ablation, complete epicardial remapping was performed after an ajmaline infusion to confirm pathological substrate abolition. In cases where residual pathological OTEGM signals were detected, additional RF applications were delivered until complete abolition was achieved. The ablation endpoint was defined as the absence of prolonged fragmented electrograms across the RVOT’s epicardial surface.

### Insights from the high-density mapping

Post-procedural remapping was performed in all cases using a standard bipolar configuration with the Advisor™ HD Grid, collecting BiEGMs along and across the splines to emulate conventional linear catheter mapping. The bipolar maps were less dense than the OT maps, with fewer acquired and displayed points (BiEGM: 734 vs. OTEGM: 1503, *p* < 0.094).

**Figure 6:**
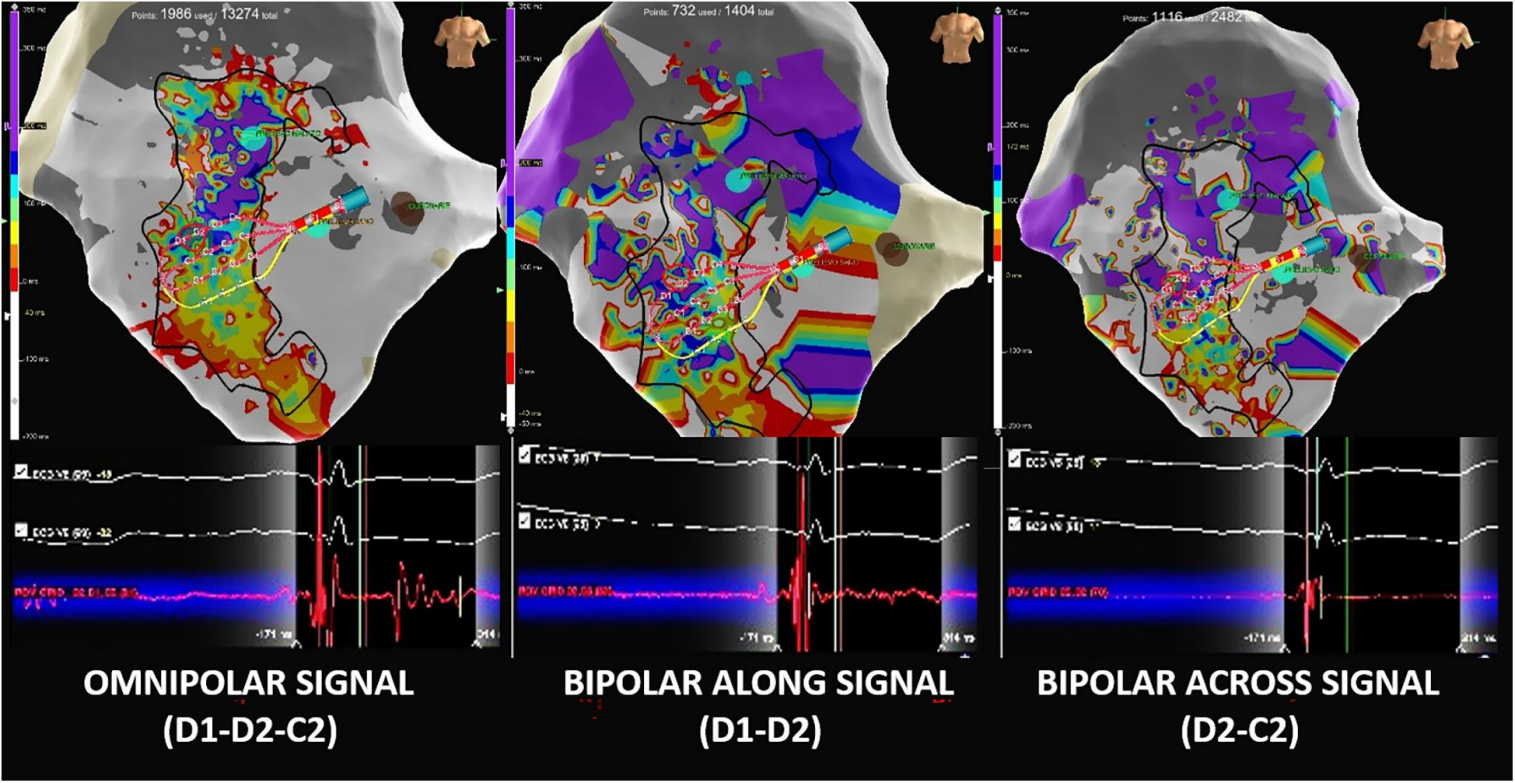
Comparison of omnipolar and bipolar electrograms. (Left) Omnipolar EGM (Advisor™ HD Grid D1-D2-C1) showing a double-component potential with a very late near-field component, which is correctly annotated by the system as the last deflection. (Center) The same cardiac beat-related bipolar EGM with an along-the-spline configuration (HD Grid D1-D2) shows a far-field late component that is missed by the late annotation due to its small signal amplitude. (RIGHT) Same cardiac beat-related bipolar EGM with an across-the-spline configuration (HD Grid D1-C1) showing only the principal ventricular component and omitting the second late component.

Standard bipolar mapping resulted in a poorer substrate delineation, requiring more human intervention due to false annotations. For several electrocardiograms, the BiEGMs failed to detect the latest and most fragmented signal components, which were clearly identified with OTEGMs (Figure 6). In addition, the OTEGM conduction vectors facilitated the identification of conduction blocks, while the wave speed maps highlighted zones of signal wavefront deceleration, data that were not available when using BiEGMs.

### Acute safety and efficacy

Complete substrate homogenization was achieved in all patients. After the post-ablation ajmaline infusions, no BrS ECG patterns reappeared. All procedures were completed without an acute adverse event.

A high impedance alert was the main cause of premature RF delivery interruption, occurring a median of 4.5 times per patient (IQR: 3.5–5.5). These interruptions were attributed to a transient loss of tip-to-tissue contact or discontinuation of saline irrigation in the open surgical field. RF delivery was resumed in all cases without clinical consequences.

The patients were discharged after a median hospitalization of 7.5 days [IQR: 6–9] with no major adverse events. Four patients experienced minor and fully reversible complications (pleural effusion and transient pericarditis) during their hospital stay.

### Follow-up

All patients completed a structured follow-up protocol with in-person evaluations at 3, 6, and 12 months post-ablation. At the 12-month visit, all patients remained in a stable sinus rhythm without spontaneous recurrence of the type I Brugada ECG pattern on standard 12-lead electrocardiography. Serial device interrogations throughout the follow-up period confirmed the absence of ventricular arrhythmias or ICD therapies. Repeat ajmaline provocation testing performed during follow-up demonstrated sustained elimination of the Brugada ECG pattern in all cases. No additional invasive electrophysiological studies were performed to reassess VT/VF inducibility.

## Discussion

### Main findings

To the best of our knowledge, this is the largest case series describing a novel hybrid mini-thoracotomy approach for substrate mapping and ablation in patients with BrS. Herein, we report on six selected patients who underwent an electrophysiologically guided surgical epicardial procedure, enabling direct visualization of the RVOT’s epicardial surface without the use of fluoroscopy or other video-assistance devices. Our preliminary findings suggest the following:

1. The approach is technically feasible for identifying and eliminating the pathological BrS substrate. After an initial learning curve, the duration of the procedure is comparable to that of other techniques of epicardial ablation.
2. The surgical approach may represent the only viable therapeutic option for patients with prior pericardial surgery who have failed percutaneous access or have extensive adhesions. The complication rate was comparable to that of other cardiac procedures requiring a mini-thoracotomy. Direct epicardial visualization enabled operator-directed biopsies from both healthy and arrhythmogenic sites, offering valuable insight into the pathophysiological mechanisms underlying BrS.
3. The high-density omnipolar mapping offered improved spatial resolution and more detailed characterization of the epicardial substrate in BrS. This technology appears to be useful for delineating the complex and heterogeneous electrophysiological features of the substrate, potentially enhancing the precision of substrate identification and ablation targeting.

### Hybrid mini-thoracotomy epicardial ablation

The hybrid mini-thoracotomy approach for epicardial ablation in Brugada syndrome has previously only been described in case reports (19, 20). The UNCOVER(BrS) study provides the first structured evidence of its feasibility, safety, and efficacy in a small but well-characterized cohort.

A rapid learning curve was observed, with procedural refinements addressing common technical challenges such as EGM noise and catheter instability (21). Procedural times, which vary widely in the literature, are reported in detail to support reproducibility (18, 21).

The procedure achieved complete substrate homogenization and ECG normalization in all patients. While the long-term impact of different ablation strategies remains to be determined, our point-by-point ablation approach resulted in electrical silence of <0.1 mV across the majority of the treated areas. This contrasts with other studies that report that RF application via the dragging technique does not significantly affect voltage amplitude or local activation times (15) or studies that lacked information on the substrate after ablation (16, 18). Our findings align with recent data showing a significant reduction in signal amplitude following ablation (22).

Recently, Namadee et al. demonstrated in a randomized clinical trial that epicardial substrate ablation significantly reduces VF recurrence in patients with symptomatic BrS who have an ICD, supporting its potential role as a first-line therapy to prevent recurrent VF in patients with symptomatic BrS (23). Although the long-term efficacy of the mini-thoracotomy ablation approach is still being investigated, early follow-up data are promising, with no episodes of arrhythmia reported in the study population. The technique also demonstrated a favorable safety profile with minor procedural complications. Despite the smaller sample size and shorter follow-up, our findings align with recent evidence (23). If these results are confirmed in larger cohorts with longer follow-up periods, the mini-thoracotomy approach may represent a viable alternative to the percutaneous technique.

### Epicardial substrate characterization

The characterization of the BrS epicardial substrate is still controversial and lacks standardization. In this study, we proposed a feasible and reproducible method for substrate assessment. OTEGMs provided additional information that was not available with standard bipolar EGMs (orientation-independent signals, maximum signal voltage, and conduction velocity) and showed improved substrate delineation performance with lower annotation errors and higher point density.

Our findings contribute to addressing the current gap in detailed electrophysiological information on surgical techniques (18, 21). The extent of the substrate still needs to be clarified. We observed significant inter-patient variability in substrate morphology and extent with the ajmaline infusion unmasking highly fragmented long-lasting EGMs, the formation of chaotic conduction with conduction block, and zones of delayed conduction. While some studies have described well-defined anatomical substrates without conduction blocks (15), others report heterogeneous activation and conduction delay (22).

The use of potential duration maps, introduced by Pappone et al., appears promising but has yet to be widely replicated (15, 17). Standardized and detailed substrate characterization is essential to improve procedural outcomes and guide ablation strategies in BrS.

### Limitations

This study was conducted at a single, highly experienced center and included only a small cohort of symptomatic patients, with limited and ongoing follow-up. These factors limit the generalizability of the findings to the broader BrS population. Anatomical variability, substrate heterogeneity, and differences in institutional expertise may significantly influence procedural outcomes.

The hybrid surgical approach is technically demanding, requiring specialized expertise and equipment that may not be widely available. A major limitation is the absence of comparative or randomized data directly comparing surgical and percutaneous epicardial ablation strategies. This lack of evidence makes it difficult to accurately assess the relative benefits, risks, and long-term outcomes of the surgical approach.

These are preliminary results and should not be considered definitive proof of efficacy; caution is warranted to avoid off-label application of the technique. Finally, the follow-up period remains short and ongoing.

## Conclusion

Substrate characterization and ablation of BrS via a hybrid mini-thoracotomy approach was shown to be technically feasible and preliminarily safe in a small cohort of highly symptomatic patients. The technique demonstrated a rapid learning curve with a notable reduction in procedural time over successive cases.

Direct epicardial mapping revealed a heterogeneous substrate with significant inter-patient variability. High-density mapping enabled more refined BrS substrate characterization and supported a patient-tailored ablation strategy.

Larger studies with extended follow-up periods are warranted to validate these initial findings and confirm the reproducibility and long-term efficacy of this technique.

## Data Availability

The raw data supporting the conclusions of this article will be made available by the authors, without undue reservation.

## References

[1] Zeppenfeld K Tfelt-Hansen J de Riva M Winkel BG Behr ER Blom NA 2022 ESC guidelines for the management of patients with ventricular arrhythmias and the prevention of sudden cardiac death. Eur Heart J. (2022) 43:3997–4126. 10.1093/eurheartj/ehac262 36017572

[2] Brugada P Brugada J. Right bundle branch block, persistent ST segment elevation and sudden cardiac death: a distinct clinical and electrocardiographic syndrome. A multicenter report. J Am Coll Cardiol. (1992) 20:1391–6. 10.1016/0735-1097(92)90253-J 1309182

[3] Brugada J Campuzano O Arbelo E Sarquella-Brugada G Brugada R. Present status of Brugada syndrome: JACC state-of-the-art review. J Am Coll Cardiol. (2018) 72:1046–59. 10.1016/j.jacc.2018.06.037 30139433

[4] Krahn AD Behr ER Hamilton R Probst V Laksman Z Han HC. Brugada syndrome. JACC Clin Electrophysiol. (2022) 8:386–405. 10.1016/j.jacep.2021.12.001 35331438

[5] Probst V Veltmann C Eckardt L Meregalli PG Gaita F Tan HL Long-term prognosis of patients diagnosed with Brugada syndrome: results from the FINGER Brugada syndrome registry. Circulation. (2010) 121:635–43. 10.1161/CIRCULATIONAHA.109.887026 20100972

[6] Kamakura S Ohe T Nakazawa K Aizawa Y Hiraoka M Ogawa S Long-term prognosis of probands with Brugada-pattern ST-elevation in leads V1–V3. Circ Arrhythm Electrophysiol. (2009) 2:495–503. 10.1161/CIRCEP.108.816892 19843917

[7] Priori SG Wilde AA Horie M Cho Y Behr ER Berul CI HRS/EHRA/APHRS expert consensus statement on the diagnosis and management of patients with inherited primary arrhythmia syndromes: document endorsed by HRS, EHRA, and APHRS in May 2013 and by ACCF, AHA, PACES, and AEPC in June 2013. Heart Rhythm. (2013) 10:1932–63. 10.1016/j.hrthm.2013.05.014 24011539

[8] Russo V Caturano A Migliore F Santoro F De Vivo S Papa AA Long-term clinical outcomes of patients with drug-induced type 1 Brugada electrocardiographic pattern: a nationwide cohort registry study. Heart Rhythm. (2024) 21:555–61. 10.1016/j.hrthm.2024.01.015 38242222

[9] Iacopino S Chierchia GB Sorrenti P Fabiano G Petretta A Cecchini F dST-Tiso interval, a novel electrocardiographic marker of ventricular arrhythmia inducibility in individuals with ajmaline-induced Brugada type I pattern. Am J Cardiol. (2021) 159:94–9. 10.1016/j.amjcard.2021.08.014 34503825

[10] Iacopino S Fabiano E Sorrenti P Cecchini F Petretta A Tripodi A Predicting ventricular arrhythmia inducibility in ajmaline-induced Brugada type I pattern: validation of the dST-Tiso interval. J Cardiovasc Electrophysiol. (2024) 35:1747–53. 10.1111/jce.16348 38923783

[11] Nademanee K Veerakul G Chandanamattha P Chaothawee L Ariyachaipanich A Jirasirirojanakorn K Prevention of ventricular fibrillation episodes in Brugada syndrome by catheter ablation over the anterior right ventricular outflow tract epicardium. Circulation. (2011) 123:1270–9. 10.1161/CIRCULATIONAHA.110.972612 21403098

[12] Brugada J Pappone C Berruezo A Vicedomini G Manguso F Ciconte G Brugada syndrome phenotype elimination by epicardial substrate ablation. Circ Arrhythm Electrophysiol. (2015) 8:1373–81. 10.1161/CIRCEP.115.003220 26291334

[13] Nademanee K Hocini M Haïssaguerre M. Epicardial substrate ablation for Brugada syndrome. Heart Rhythm. (2017) 14:457–61. 10.1016/j.hrthm.2016.12.001 27979714

[14] Nademanee K Raju H de Noronha SV Papadakis M Robinson L Rothery S Fibrosis, connexin-43, and conduction abnormalities in the Brugada syndrome. J Am Coll Cardiol. (2015) 66:1976–86. 10.1016/j.jacc.2015.08.862 26516000 PMC4631798

[15] Pappone C Brugada J Vicedomini G Ciconte G Manguso F Saviano M Electrical substrate elimination in 135 consecutive patients with Brugada syndrome. Circ Arrhythm Electrophysiol. (2017) 10:e005053. 10.1161/CIRCEP.117.005053 28500178

[16] Nademanee K Chung FP Sacher F Nogami A Nakagawa H Jiang C Long-term outcomes of Brugada substrate ablation: a report from BRAVO (Brugada ablation of VF substrate ongoing multicenter registry). Circulation. (2023) 147:1568–78. 10.1161/CIRCULATIONAHA.122.063367 36960730

[17] Santinelli V Ciconte G Manguso F Anastasia L Micaglio E Calovic Z High-risk Brugada syndrome: factors associated with arrhythmia recurrence and benefits of epicardial ablation in addition to implantable cardioverter defibrillator implantation. Europace. (2024) 26:1–13. 10.1093/europace/euae019 PMC10824473 38252933

[18] Salghetti F de Asmundis C Sieira J Coutino HE Abugattas JP Varnavas V Hybrid thoracoscopic epicardial ablation of right ventricular outflow tract in patients with Brugada syndrome. Heart Rhythm. (2019) 16:879–87. 10.1016/j.hrthm.2018.12.026 30594641

[19] Iacopino S Cecchini F Tripodi A Sorrenti P Fabiano G Petretta A. Epicardial multisite conduction blocks detected by equispaced electrode array and omnipolar technology in Brugada syndrome. HeartRhythm Case Rep. (2023) 9:12–6. 10.1016/j.hrcr.2022.09.015 36685695 PMC9845549

[20] Cecchini F Iacopino S Tripodi A Sorrenti P Fabiano G. Hybrid minithoracotomy approach for zero-fluoroscopy epicardial ablation of the arrhythmogenic substrate in Brugada syndrome. HeartRhythm Case Rep. (2022) 8:562–6. 10.1016/j.hrcr.2022.05.012 35996709 PMC9391404

[21] Eltsov I Pannone L Ramak R Monaco C Della Rocca DG Bala G 3D mapping challenges in hybrid video-assisted thoracoscopic surgical ablation of Brugada syndrome. Interdiscip Cardiovasc Thorac Surg. (2023) 37:ivad160. 10.1093/icvts/ivad160 37756702 PMC10541674

[22] Li L Ding L Zhou L Wang Y Zhang Y Liu Y Outcomes of catheter ablation in high-risk patients with Brugada syndrome refusing an implantable cardioverter defibrillator implantation. Europace. (2024) 26:1–10. 10.1093/europace/euad318 PMC10754161 37889958

[23] Nademanee K Wongcharoen W Chimparlee N Chokesuwattanaskul R Annueypol M Phusunti K Brugada syndrome ablation for the prevention of ventricular fibrillation episodes (BRAVE). Heart Rhythm. (2025) 22:1975–83. 10.1016/j.hrthm.2025.04.033 40294736

